# Newborn feeding recommendations and practices increase the risk of development of overweight and obesity

**DOI:** 10.1186/s12887-020-1982-9

**Published:** 2020-03-04

**Authors:** Brittany Watchmaker, Bridget Boyd, Lara R. Dugas

**Affiliations:** 1 Loyola University Chicago Medical Center, Chicago, Illinois USA; 2 Department of Pediatrics, Loyola University Chicago Medical Center, Chicago, Illinois USA; 3 Department of Public Health, Loyola University Chicago Medical Center, Chicago, Illinois USA

**Keywords:** Obesity, Formula, Baby friendly, Overfeed, Over-nutrition, Metabolic programming, Intervention, Global health

## Abstract

**Background:**

The prevalence of obesity among infants less than 2 years of age has increased by more than 60% over the last three decades. Obese infants and toddlers are at an increased risk for staying overweight into adolescence and adulthood. Metabolic programming has been demonstrated in animal models whereby early life feeding habits result in life-long changes in hormone balance and metabolism. Our study explores if newborn over-nutrition on the first day of life (DOL1) is associated with risk for future overweight and obesity in childhood.

**Methods:**

Retrospective chart data was collected for full term formula-fed infants born between January 2008 and December 2012 who continued care at the same institution. Data included the volume of formula (ml) consumed on DOL1 as well, as subsequent yearly BMI measures from well child checkups (WCC). Overfeeding was categorized as any feed greater than or equal to 30 ml on any of the first seven feeds while controlling for birth weight.

**Results:**

The final data set included 1106 infants (547 male; 559 female). 1023 of the 1106 newborns (93%) were overfed at least once during DOL1, while 789 of 1106 (71%) were overfed 3 or more times during their first 7 feeds. After adjusting for birth weight, infants who were overfed 5 of the first 7 feeds were 5 times as likely to be overweight or obese at their 4th year well child check-up (*p* < 0.05) compared to children not overfed. Infants who were overfed on all 7 of their first 7 feeds were 7 times more likely to be overweight or obese at their 4th year WCC (*p* = 0.017).

**Conclusions:**

Infants overfed on DOL1 were significantly more likely to be overweight or obese at their 4th year WCC, compared to infants not overfed on their first day of life. Newborn families may benefit from counseling regarding age-appropriate volumes of formula during this critical time period. Future studies will aim to look at effect of implementation of smaller feeding bottle size on reducing overfeeding practices and future risk of overweight and obesity.

## Background

Obesity is a serious public health problem with more than 200 million overweight and obese children worldwide [1]. The prevalence of obesity among infants less than two years of age has increased by more than 60% over the last three decades, and obese infants and toddlers are at an increased risk for staying overweight into adolescence and adulthood [1]. While obesity is a multi-factorial disease, identifying the largest contributors remains a priority. The early-life period has been increasingly studied as the most optimal time to intervene in this epidemic. It is suggested that the early life period is particularly susceptible to environmental “programming” effects that have been evidenced to remain present much later in life [2, 3]. Specifically, rapid weight gain early in life due to improper feeding practices has been shown to be a risk factor for obesity later in childhood [3, 4]. While breastfeeding has been shown to be protective against diseases and conditions including childhood obesity, the volume of milk being consumed by the infant is difficult to measure. On the other hand, among families who need or chose to formula feed, the volume consumed is a modifiable risk factor that could lessen the predisposition to become overweight and obese [5].

Overeating may be learned in infancy, even as early as the first day of life, or Day Of Life 1 (DOL1) [6, 7]. In rodent studies, overfeeding as early as the second day of life has been shown to lead to brown adipose tissue hypo-activity. This supports the early programming hypothesis for obesity risk, as brown adipose tissue is protective against obesity through energy expenditure needed for thermogenesis [8]. Additionally, early overfeeding in female rats has been shown to have long-term dysregulatory effects in the ghrelin signaling pathway [9]. Other factors such as protein content of formula have been shown to affect future body weight composition well into childhood [10].

Guidelines for the proper volume of formula a newborn should receive in the first days of life include graphic depictions including that of a shooter marble (5-7 ml) on DOL1, a ping pong ball (22–27 ml) on DOL3, and an extra-large chicken egg (60–81 ml) on DOL10 [11]. These physiologic capacities and corresponding feeding volumes, however, are not widely enforced, and consequential over-nutrition may set newborns on a trajectory of overweight and obesity [7]. The physiologic capacity of a newborn stomach can be defined as the volume that the stomach can comfortably hold without causing discomfort or high intra-gastric pressures. This is the optimal volume for a feed on DOL1, the amount the stomach *should* hold. The anatomic capacity of a newborn stomach, on the other hand, can be defined as the volume the newborn stomach *can* hold, after stretching and reaching high intra-gastric pressures and discomfort [12].

To date, only a handful of studies have explored this topic. One such meta-analysis looked at 6 separate studies and concluded the newborn stomach anatomic capacity to be 20 mL on DOL1, with an optimal feeding frequency of every 1-h [13]. Another study looked at post-mortem data, where newborn stomachs were filled to high pressures [12]. They found that almost regardless of birth weight, filling the newborn stomach to 30-35 mL causes high pressures resulting in significant gastric discomfort [12]. Finally, a third study found the pressure in a newborn stomach to double at a filling volume of 20 mL [14]. These recordings stopped at 30 mL which correlated with the pain pressure threshold of an adult. Based on this literature review, for the purposes of analysis, we safely defined an overfed meal as any feed greater than or equal to 30 mL on DOL1. Formula companies, however, recommend 2–3 oz (60–90 mL) of formula every 3 h in the newborn period. These recommendations on feeding practices in the impressionable early life period could have serious consequences and contribute to overweight and obesity later in life.

We therefore sought to explore the association between infant over-nutrition on DOL1 and its associations with overweight and obesity later in childhood. We hypothesized that the volume of formula consumed on an infant’s DOL1 is a risk factor for overweight and obesity later in life.

## Methods

### Study design

The study was approved by the Loyola University Chicago Institutional Review Board, and marked except (LU209901). A retrospective chart review was performed using the Loyola University Medical Center (LUMC) electronic healthcare records for children born between January 1st 2008 and December 31st 2012. Inclusion criteria included exclusively formula fed infants gestational age of 35 (0/7) to 42 (0/7) who continued their pediatric care at Loyola and had at least 2 Body Mass Index (BMI) measurements from well-child check visits (WCC), up to the age of 5 years. Exclusion criteria included inpatient hospital stay longer than 5 days, birth weight less than 2.2 kg, and any Neonatal Intensive Care Unit (NICU) stay greater than 2 days to limit our population to presumed healthy newborns. We therefore, set out to analyze full term, formula fed infants at LUMC born between 1/1/2008 and 12/31/2012, with complete DOL1 formula feeding data. Other variables included gestational age, birth weight, length, dichorionic weight, Date Of Birth (DOB), maternal age, zip code, insurance, ethnicity, delivery method, and importantly the volume in ml of all inpatient feeds (Table 1). An overfed meal was defined as the consumption of any meal volume equal to or above 30 ml on DOL1. We restricted our analysis to the first 7 feeds on DOL1, because this represented the average number of feeds a newborn in our cohort received on DOL1. Formula feeding data (volume of formula consumed by the newborn) was collected based on the amount the newborn consumed in each feed. The newborn was given a 60 cc bottle of ready feed formula (Similac 20 kcal), and after each feed, nursing team members would record (in mL) the volume of the feed in our Electronic Medical Record (EMR). We used the CDC’s definition of overweight and obesity, definition underweight as < 5th percentile, normal weight as 5th to <85th percentile, overweight as 85th to <95th percentile, and obese as 95th percentile or greater.

**Table 1:** Demographic information collected for our population included gender, race, ethnicity, length of stay, delivery type, birth weight, and volume of formula consumed on each feed in DOL1

Demographic	Male	Female	Total
Gender	547	559	1106
Race	545	558	1103
*-Asian*	9 (2%)	11 (2%)	20
*-Black*	223 (41%)	200 (36%)	423
*-Hispanic*	14 (3%)	19 (3%)	33
*-Multicultural*	0 (0%)	3 (1%)	3
*-Other*	98 (18%)	117 (21%)	215
*-Not indicated*	5 (1%)	2 (.4%)	7
*-White*	196 (36%)	206 (37%)	402
Ethnicity	544	559	1103
*-Hispanic*	138 (25%)	172 (31%)	310
*-Non-Hispanic*	402 (73%)	381 (68%)	783
*-Unknown*	4 (1%)	6 (1%)	10
Length of Stay			
*-1 day*	123 (22%)	130 (23%)	253
*-2 days*	292 (53%)	302 (54%)	594
*-3 days*	108 (20%)	99 (18%)	207
*-4 days*	18 (3%)	24 (4%)	42
*-5 days*	6 (1%)	4 (1%)	10
Delivery Type			
*-C-Section*	196 (36%)	204 (36%)	400
*-Non-C-Section*	362 (66%)	374 (67%)	736
Birth weight (oz.)	117.49	133.71	125.68
Overfeeds in DOL1 (Feeds ≥ 30 mL)			
0	45 (8%)	38 (7%)	83
1	51 (9%)	48 (9%)	99
2	67 (12%)	68 (12%)	135
3	87 (16%)	93 (17%)	180
4	93 (17%)	76 (14%)	169
5	82 (15%)	84 (15%)	166
6	69 (13%)	87 (16%)	156
7	53 (10%)	65 (12%)	118

### Statistics

Based on a literature review, we defined an overfed meal as a formula feed greater than or equal to 30 mL on an infant’s DOL1, and we included the first seven feeds as counting towards DOL1 feeds. Follow-up WCC BMI data was collected and the children were categorized as either underweight, normal weight, overweight, or obese at each of their yearly visits (Table 2) according to the CDC guidelines for weight categories.

**Table 2:** Follow-up WCC BMI data was collected and the children were categorized as either underweight, normal weight, overweight, or obese at each of their yearly visits according to the CDC guidelines. Twenty five percent of our cohort presented as overweight or obese at the 2nd year WCC, 25% presented as overweight or obese at their third year WCC, 33% at their 4th year WCC, and 35% at their 5th year WCC

Weight Category	Well Child Checkup 2	Well Child Checkup 3	Well Child Checkup 4	Well Child Checkup 5
Underweight (< 5%)	28	21	11	10
Normal weight (5–84.9%)	236	179	151	118
Overweight (85–94.9%)	25	29	34	28
Obese (> 95%)	26	38	44	40

Data were tabulated to summarize means, and standard deviations (SD). We used multivariate analysis logistic regression, to explore the association between overfed newborns on DOL1 and risk for overweight and obesity later in childhood, adjusting for co-variates including maternal and socio-economic factors. An alpha *p*-value of 0.05 was used to denote statistical significance. We used STATA (v.12, College Station, TX) for statistical analysis to tabulate frequency of overfeeds among our cohort.

## Results

### Participant characteristics (Table 1)

The final sample included 1106 formula fed infants, of which 547 were male and 559 were female (Table 1). 77% of the population had a length of stay (LOS) of one or 2 days, while all had LOS 5 days or less. 36% of the population was delivered by cesarean-section while 64% were non-cesarean section birth. Twenty five percent of our cohort presented as overweight or obese at the 2nd year WCC, 25% presented as overweight or obese at their third year WCC, 33% at their 4th year WCC, and 35% at their 5th year WCC. These numbers are in line with current overweight and obese prevalence data among United States children.

### Overfeeding practices

Using the overfeeding definition of any feed greater than or equal to 30 mL, we found that 99 of the 1106 (9%) newborns in our cohort were overfed once in their DOL1, 135 were overfed twice (12%), 180 were overfed 3 times (16%), 169 were overfed 4 times (15%), 166 were overfed 5 times (15%), 156 were overfed 6 times (14%), and 118 were overfed all 7 times of their first 7 feeds (11%) (Table 1). Only 83 of the 1106 (8%) newborns in our study were not overfed any of their feeds on DOL1 Accordingly, 71% (789/1106) of the newborns had been overfed 3 or more of their first 7 feeds (Table 1).

### Risk for overweight and obesity from overfeeding

After adjusting for birth weight, infants who were overfed 5 of the first 7 feeds were 5.19 times as likely to present as overweight or obese at their 4th year WCC (*p* = 0.050), while infants who were overfed all 7 of the first 7 feeds of life were 7.22 times as likely to be overweight or obese at their 4th year WCC compared to children not overfed on their first day of life (*p* = .017) (Table 3).

**Table 3:** Number of overfeeds on DOL1 and corresponding odds ratio of overweight or obese at Well Child Check-up age 2, 3, 4, and 5 years of age (after adjusting for birth weight)

	WCC2		WCC3		WCC4		WCC5	
# Of Overfeeds on DOL1	Odds Ratio	*p*-value	Odds Ratio	*p*-value	Odds Ratio	*p*-value	Odds Ratio	*p*-value
1	.98	.979	.37	.266	**2.56**	**.286**	1.14	.921
2	1.07	.936	.62	.628	**3.54**	**.140**	1.00	.997
3	1.39	.658	.58	.256	**4.75**	**.060**	1.82	.619
4	.62	.555	.42	.162	**2.41**	**.317**	1.10	.937
5	1.19	.814	.50	.666	**5.19**	**.050**	1.38	.791
6	1.15	.855	1.44	.120	**6.40**	**.025**	1.01	.993
7	1.63	.514	.77	.602	**7.22**	**.017**	3.27	.339

### Birth weight and risk of overfeeding

**Fig. 1:**
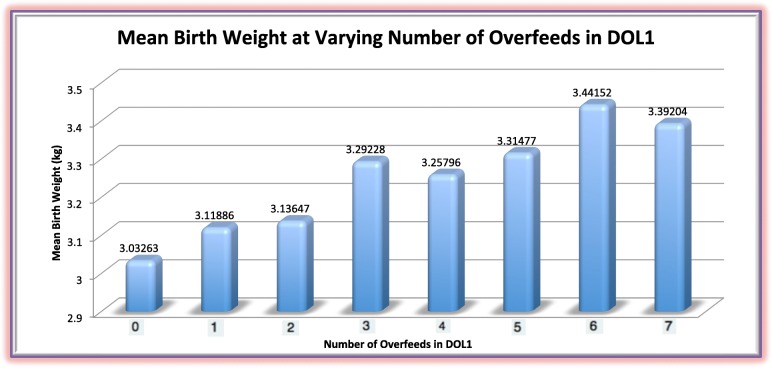
This figure shows that for every extra overfeed in DOL1, the birth weight increased by 54 g *p* < .001

Figure 1 presents the relationship between mean birth weight and number of overfeeds on DOL1. Birth weight is significantly positively correlated to increased number of overfeeds in DOL1 (*p* < .001). For every additional overfeed on DOL1, the mean birth weight increased by 54 g (*p* < .001) (Table 4).

**Table 4:** This table shows well child check-up age (2, 3, 4, and 5 year WCC) and the corresponding odds ratios for presentation as overweight or obese when overfed 4 or more times in DOL1, after adjusting for birth weight

Well Child Check-Up	Odds Ratio for Overweight or Obesity when overfed 4 or more times in DOL1	*p*-value
2	.974	.936
3	1.24	.484
4	1.66	.079
5	1.01	.987

## Discussion

We found that overfeeding infants 5 or more times in the first day of life significantly increases the risk for being overweight or obese at a child’s 4th year well child check. Although current guidelines for the proper volume of formula a newborn should receive are available, these guidelines are not widely enforced or followed by hospital personnel and new parents, placing the newborn at for future overweight or obesity. Furthermore, formula companies advise newborns consume 2–3 oz. (60–90 ml) every 3 h in their first weeks of life, which is significantly greater than what the literature recommends in the first day of life [12, 14, 15].

A longitudinal study based in Australia by Oddi et al. followed 2868 live births and the subsequent early feeding practices during their first months of life, and found that early feeding practices have a significant effect on long-term BMI and adiposity [16]. This study suggests that rapid early growth in life and early BMI trajectory is a risk factor for continued and later increased adiposity [16]. Although this study mainly focused on formula feeding as compared to breastfeeding resulting in more rapid weight gain during infancy, the same idea of rapid weight gain due to overfeeding may be able to be applied as elucidated in our study.

Additionally, Du, Hosada, Umekawa et al. investigated the effects of early overfeeding of mice pups of the C57BL/6 N breed, the most commonly used general purpose strain of mice. Pups that were fed a high fat diet, had on postnatal weight gain in comparison to pups that were fed under normal conditions [17]. The effects of these different feeding conditions on glucose metabolism were measured on post-natal day 7, 14, and 21 [17]. Unsurprisingly, the weight gains of the overfed and pups fed a high fat diet were 1.2 times greater than the control diet pups [17]. Interestingly, however, the overfed pups were found to have both higher blood glucose and serum insulin levels compared to that of the control group of pups, while the high fat diet pups only had higher blood glucose levels than the control [17]. Insulin resistance was also found in the overfed and high fat diet pups.

A similar investigation explored underfed, typically fed, and overfed rats and determined that by as early as day 15 of life, changes in metabolic pathways of these rats had already formed and were found to persist into adulthood in a way that significantly increased the chances for overweight and obesity [3]. This study also suggests that that hyperinsulinemia induced by the overfeeding has critical effects on the developing brain and may also contribute to later overweight and obesity. Overall, these studies support the idea that early environmental stimuli can have lasting metabolic changes and promote a trajectory of overweight or obesity [3].

Guidelines suggesting that newborn infants receive no more than 30 mL per feed during DOL1 may be a critical addition to the prevention of overweight and obesity in children and adolescents. We hypothesized that feeding habits demonstrated in the first day of life could be indicative of future feeding habits that extend into childhood and could be a risk factor for becoming overweight or obese.

The Baby-friendly Hospital initiative (BFHI) was created in 1991 by the WHO and UNICEF to promote and support breastfeeding. Currently, over 152 countries are compliant with this initiative and have earned Baby-friendly designation [18]. The institution in the current study adopted this mission and earned Baby-Friendly designation in 2012. Although this initiative promotes exclusive breastfeeding for the first six months, it also promotes healthy feeding practices among formula fed newborns if the mother is unable or chooses not to breastfeed. As part of this initiative, our institution noticed the pattern of overfeeding and introduced a potential solution. Historically, mothers of formula fed newborns were given a 60 mL ready-made bottle of formula with little instruction on the proper volume to feed. Many families were feeding the full 60 ml at each feeding often resulting in frequent emesis and baby discomfort. As previously discussed, this volume far exceeds the physiologic capacity of the newborn stomach on DOL1. In an attempt to improve this issue our institution now gives families specific instructions on proper volumes to feed on each day of life and provides a separate bottle to pour formula into before beginning a feed.

Our study has several limitations. Firstly, we only included babies with complete DOL1 formula feeding data, and who had at least 2 complete well child visits during the study period. Data from children who followed up at outside institutions could not be followed. Secondly, we did not have a control group, or include breast fed babies due to inability to track specific volumes. Finally, obesity is a multifactorial disease with many contributing factors such as genetics, family lifestyle, culture, inactivity, unhealthy diet, medical problems and social or economic issues. While we attempted adjust our analyses for these covariates, it is likely, that not all the covariance were removed. For example, we did not include factors such as maternal BMI and weight gain during pregnancy which may be of importance for risk of future overweight or obesity.

Our cohort included newborns who were born prior to the hospitals adoption of the BFHI, and introduction of separate bottles for portion control. Future studies should investigate whether this initiative has had an impactful reduction in newborn over-nutrition and subsequent reduced risk for overweight and obesity at follow-up outpatient care visits. Other future directions of this ongoing study includes looking at feeding trends in general over time from 2007 to current 2018 data. We hope to study feed intervals more closely as a risk factor for obesity later in life. For example, some newborns may have not met our criteria for an overfeed, however; may have been overall overfed due to increased frequency of feeds. Finally, we hope to follow our cohort over time as obesity may present at later ages.

In conclusion, we have shown that overfeeding on the first day of life is an independent risk factor for the development of overweight and obesity. Reinforcing newborn feeding guidelines to hospital personnel and new parents may be a critical component in confronting the childhood and adolescent obesity epidemic.

## Data Availability

The datasets analyzed during the current study are available from the corresponding author on reasonable request.

## Abbreviations

BMI: Body Mass Index
DOL1: First day of life
EMR: Electronic Medical Record
LUMC: Loyola University Medical Center
